# The Effect of Micro-Inertia and Flexoelectricity on Love Wave Propagation in Layered Piezoelectric Structures

**DOI:** 10.3390/nano11092270

**Published:** 2021-08-31

**Authors:** Olha Hrytsyna, Jan Sladek, Vladimir Sladek

**Affiliations:** 1 Institute of Construction and Architecture Slovak Academy of Sciences, Dúbravská Cesta 9, 84503 Bratislava, Slovakia; Jan.Sladek@savba.sk (J.S.); vladimir.sladek@savba.sk (V.S.); 2 Center of Mathematical Modeling of Pidstryhach Institute for Applied Problems of Mechanics and Mathematics, National Academy of Sciences of Ukraine, Dudajev Str. 15, 79005 Lviv, Ukraine

**Keywords:** love waves, strain gradient theory, micro-inertia effect, piezoelectricity, flexoelectricity, dispersion relation

## Abstract

The non-classical linear governing equations of strain gradient piezoelectricity with micro-inertia effect are used to investigate Love wave propagation in a layered piezoelectric structure. The influence of flexoelectricity and micro-inertia effect on the phase wave velocity in a thin homogeneous flexoelectric layer deposited on a piezoelectric substrate is investigated. The dispersion relation for Love waves is obtained. The phase velocity is numerically calculated and graphically illustrated for the electric open-circuit and short-circuit conditions and for distinct material properties of the layer and substrate. The influence of direct flexoelectricity, micro-inertia effect, as well as the layer thickness on Love wave propagation is studied individually. It is found that flexoelectricity increases the Love-wave phase velocity, while the micro-inertia effect reduces its value. These effects become more significant for Love waves with shorter wavelengths and small guiding layer thicknesses.

## 1. Introduction

The first theoretical studies on shear surface waves that propagate in a layered structure were done by Love [1] who showed that such waves can propagate in an isotropic layer deposited on an isotropic substrate if the velocity of shear wave in the layer is slower than that in the substrate. Nowadays, these horizontal surface shear waves are referred to as Love waves. Since the surface waves are effectively used in practice [2,3], the propagation of shear horizontal waves of Love-type attracts much attention and interest of researchers. A lot of scientists focused on the investigations of Love wave behavior in crystals of different classes. Dispersion relations of Love-type waves were numerically found for layered structures consisting of the class-23 cubic piezoelectric crystals in [4]. The Love waves generating in an elastic layer SiO_2_ deposited on a piezoelectric substrate ST-90X were analytically studied by Liu and He [5]. The investigation of surface waves in structures with more than one layer was a subject of many papers (see, for example, [6,7,8]). In [7] the behavior of elastic waves in the three-layered piezoelectric media with inhomogeneous initial stresses was analyzed through the method of transfer matrix. Propagation of electro-elastic surface Love waves in a piezoelectric substrate of crystal class 6, 4, 6 mm, and 4 mm covered with one or two layers was considered by Danoyan and Piliposian [9,10]. In [10], the effect of the second (conducting or dielectric) layer on the propagation behavior of Love wave in a three-layered structure was studied.

Manna et al. [11] investigated the propagation of Love wave in a piezoelectric layer overlying an inhomogeneous half-space. Du with coworkers [12] and later Eskandari and Shodja [13] considered the propagation behavior of Love waves in functionally graded piezoelectric materials. Kiełczynski et al. [14] presented a theoretical study of ultrasonic Love waves in nonhomogeneous functionally graded semi-infinite elastic half-space whose elastic properties vary monotonously with the distance from the substrate surface.

The effect of the viscous dissipation on wave propagation in layered ‘viscoelastic—piezoelectric’ and ‘piezoelectric—viscoelastic’ structures was analytically studied in papers [15,16]. Recently, much attention was paid to the investigation of the effect of initial stress on Love wave behavior in piezoelectric structures [7,17,18], magnetoelectroelastic continua [19], porous media [20], and granular materials [21]. The propagation of Love waves in polarized ceramics using equations of linear piezoelectromagnetism was discussed in [19,22,23].

The Love wave behavior in layered structures with the piezoelectric thin film deposited on the elastic substrate is theoretically examined by Zhang et al. [24]. The surface effects are considered there too. If the surface effects are considered, the frequency dispersion of Love waves is strongly dependent on film thickness if the guiding layer thickness reduces to nanometers. They also showed that phase velocities of all dispersion modes increase with decreasing of the film thickness. Hence, when the guiding layer is nano-sized the surface effects become very dominant. This means that when the thickness of the guiding layer is comparable to the material internal length scale parameter, the non-classical continuum theories with a size effect should be applied to get a physically appropriate result.

One of the first publications on the study of Love wave behavior using the higher-grade theory was the work by Majorkowska-Knap and Lenz [25]. The Mindlin theory of piezoelectricity with polarization gradient is applied there to study Love waves in the case of propagation along a centrosymmetric, isotropic, dielectric layer attached to an isotropic elastic half-space. The conditions for the existence of these waves were analyzed. Special attention was paid to the study of the influence of the material and geometric parameters of a layered structure on the phase velocity of Love wave. From the classical theory it follows that when the velocity $c_{sh}^{h}$ of transverse bulk waves in the substrate is larger than that in the layer (i.e., $c_{sh}^{h}>c_{sh}$), surface waves of arbitrary angular frequency can propagate through the medium. Majorkowska-Knap and Lenz found that, in contrast to the classical case, Love waves can also exist in a dielectric layer of Mindlin’s type under the non-classical condition $c_{sh}^{h}<c_{sh}$ for the ‘cut-off frequencies’. Based on the Mindlin gradient theory, they established the corresponding relation for the mentioned cut-off frequencies.

Recently, the strain gradient theory of electroelastic media with flexoelectricity was employed to solve the Love wave propagation problems [26]. They considered the effect of the high-order electric quadrupoles, in addition to the strain gradients. The strain gradient and electric quadrupoles are linked with direct and converse flexoelectric effects. Yang with co-authors [26] showed that flexoelectricity can greatly influence the phase velocity of surface waves and therefore it plays an important role in the studies of these waves. They revealed that the effect of flexoelectricity depends on the thickness of the guiding layer and on the values of flexoelectric coefficients. They also found that in contrast to the classical theory if the flexoelectricity is taken into account the ‘cut-off wave number’ can emerge when the real part of phase velocity exceeds the shear bulk wave velocity in the substrate. They concluded that flexoelectricity should not be neglected in nano-scale piezoelectric structures.

Two years later, making use of the strain gradient piezoelectricity, Singhal et al. [27,28] analytically investigated Love-type wave vibrations in a piezoelectric thin film overlying the pre-stressed elastic plate of SiO_2_. Using the material coefficients for PZT-2, PZT-4, PZT-5H, LiNbO_3_, and BaTiO_3_, they analyzed the effect of material properties of guiding layers on phase velocity. They also concluded that flexoelectricity plays an important role in layered structures at the micro- and nano-scales.

Studies of Love waves within the strain gradient theory of piezoelectricity have shown that the flexoelectric effect is more pronounced for sufficiently large wave numbers. Besides, many researchers have revealed that for high-frequency waves it is very important to consider the micro-inertia effect [29,30,31,32,33]. Ottosen et al. [34], Georgiadis and Velgaki [30] observed that in some cases the classical continuum theories are not capable of fully and appropriately describing the electromechanical behavior of short-length waves and the micro inertia effect has to be considered. Using the couple stress theory with micro inertia term, the elastodynamic fields of an anti-plane shear wave scattered by a micro-/nano-fiber embedded in an infinite matrix have been investigated by Shodja et al. [31]. They show that, in contrast to the classical elasticity and couple stress theory (without micro inertia), which give incorrect results at high frequencies with wavelengths comparable to the material internal length scales, the dispersion curves predicted within the couple stress theory with micro-rotatory inertia term are in agreement with experimental works. They have found that the effect of micro-rotatory inertia term is noticeable at higher frequencies. Hu with co-authors [35] studied the longitudinal wave propagation behavior in a semi-infinite elastic dielectric medium. In this work, the effect of flexoelectricity, micro-inertia, and strain gradient elasticity was taken into consideration. The authors found that the phase velocity and group velocity predicted by the strain gradient elasticity without considering the micro-inertial are unbounded and thus physically unacceptable. Yang et al. [33] analyzed the influences of flexoelectricity, strain gradient elasticity, micro-inertia effect, and surface phenomena on Rayleigh waves in a homogeneous centrosymmetric half-space. They reported that strain gradient elasticity and surface elasticity can increase the phase velocity, while the micro-inertia effect decreases the phase velocity. These studies revealed that the effect of the micro inertia term is more noticeable at higher frequencies.

Although the flexoelectric effect on wave propagation has been investigated in the above-mentioned literature, the complex influence of the flexoelectricity and micro-inertia effect on the Love wave propagation in piezoelectric materials has not been reported so far. Thus, the goal of this paper is to study both the flexoelectric and micro-inertia effect on the Love wave propagating in a nano-sized guiding layer rigidly bonded to a piezoelectric substance.

The paper is organized as follows. In Section 2, the generalized linear governing equations and boundary conditions of strain gradient theory are used to study Love waves which propagate along the free surface of the semi-infinite piezoelectric substrate covered with a thin guiding flexoelectric layer. Along with flexoelectricity, the governing equations also take the micro-inertia effect and piezoelectric properties of the substrate into account. The analytical solution to the formulated boundary-value problem and the general dispersion equation for Love waves in the mentioned layered structure are obtained for the electric open-circuit and short-circuit conditions. In Section 3, the numerical results and a detailed analysis of the phase velocity of Love wave are provided for material combinations ‘PZT-5H—LiNbO_3_′ and ‘PZT-5H—BaTiO_3_′. The influence of the micro-inertia characteristic length, flexoelectric coefficients, guiding layer thickness, and the substrate piezoelectricity constant on Love waves is discussed. The main conclusions are reported in the final Section 4.

## 2. Formulation and Theoretical Treatment of the Problem

**Figure 1:**
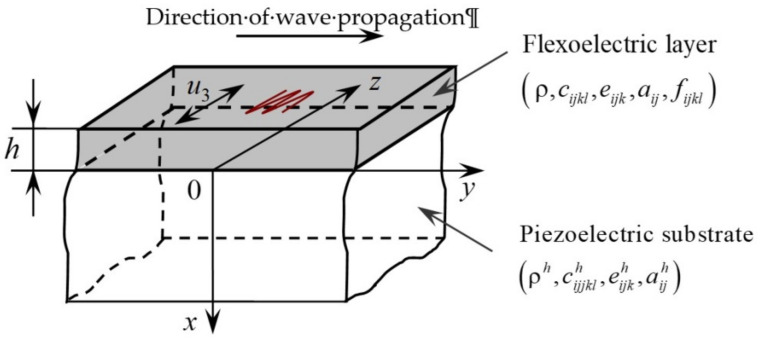
Layered piezoelectric structure and choice of the coordinate system.

The propagation of Love waves is studied in a layered piezoelectric structure. A thin flexoelectric layer −h≤x≤0 is overlaying the piezoelectric half-space x≥0. The domain of x≤−h is an air or vacuum in contact with the traction-free surface of the layer. The flexoelectric layer is rigidly linked to the semi-infinite piezoelectric substrate. The flexoelectric properties of the dielectric substrate are omitted. A rectangular Cartesian coordinate system (*x*, *y*, *z*) is chosen in such a way that the *y*-axis is parallel to the direction of Love-wave propagation, and the x-axis is vertical to the surface of the substrate. The piezoelectric structure and the relevant coordinate axes are given in Figure 1. We suppose that the upper surface of the flexoelectric layer (x=−h) is traction-free and can be either free of electric charge or grounded (with vanishing electric potential).

### 2.1. Basic Equations for Flexoelectric Continua with Micro-Inertia Effect

The effect of material microstructure on wave behavior cannot be described by the classical theory, but the higher-order or generalized theories of elasticity possessing internal length scale(s) can be used. The strain gradient theory [36] is applied here to investigate Love waves in the nanoscale flexoelectric layer deposited on the elastic substrate. Constitutive equations for a piezoelectric material with the direct flexoelectric effect can be written as [26]

(1) $$\sigma_{ij}=c_{ijkl}\varepsilon_{kl}-e_{kij}E_{k},$$

(2) $$\mu_{jkl}=-f_{ijkl}E_{i},$$

(3) $$D_{i}=a_{ij}E_{j}+e_{ijk}\varepsilon_{jk}+f_{ijkl}\eta_{jkl}.$$

Here, symbols $\sigma =\left\{\sigma_{ij}\right\}$ and $\mu =\left\{\mu_{ijk}\right\}$ are used to denote the stress tensor and the higher-order stress tensor, respectively, $\varepsilon =\left\{\varepsilon_{kl}\right\}$ is the strain tensor, $\eta =\left\{\eta_{jkl}\right\}$ is the strain-gradient tensor, $E=\left\{E_{k}\right\}$ and $D=\left\{D_{i}\right\}$ denotes the electric intensity and electric displacement vectors, $c=\left\{c_{ijkl}\right\}$, $e=\left\{e_{kij}\right\}$, and $a=\left\{a_{ij}\right\}$ represent the elastic, piezoelectric, and permittivity constants, and $f=\left\{f_{ijkl}\right\}$ is the fourth-rank tensor of flexoelectric constants.

Note that the strain gradient term in constitutive Equation (2) is neglected for simplicity as was done in works [26,37].

Within the usual assumption of small strain theory, the kinetic equations are defined as

(4) $$\varepsilon_{ij}=\frac{1}{2}\left(u_{i,j}+u_{j,i}\right),\;\;\;\;\;\;\eta_{ijk}=\varepsilon_{ij,k}=\frac{1}{2}\left(u_{i,jk}+u_{j,ik}\right),$$

where $u_{i}$ is the component of the displacement vector, and comma stands for partial differentiation with respect to the indicated space coordinate. The strain-gradient tensor η, piezoelectric constants tensor e, and tensor of flexoelectric constants f obey the symmetry property defined as $\eta_{ijk}=\eta_{jik}$, $e_{ijk}=e_{ikj}$, and $f_{ijkl}=f_{ikjl}$.

Being interested in mechanical excitations and taking into account the fact that characteristic frequency for electromagnetic fields is significantly higher than that for elastic fields, one can consider a quasi-static approximation for the electromagnetic fields. Then, in considered materials, the magnetic field is absent and the electric field intensity can be described by the gradient of a scalar electric potential $\varphi_{e}$ [38]:

(5) $$E_{i}=-\varphi_{e,i}.$$

To incorporate the micro-inertia effect into mathematical model, the following expression for the kinetic-energy density is considered [39]

$$K=\frac{1}{2}\rho {\dot{u}}_{i}{\dot{u}}_{i}+\frac{1}{2}\rho l_{1}^{2}{\dot{u}}_{i,j}{\dot{u}}_{i,j}.$$

Here, ρ is the mass density in the layer, $l_{1}$ is defined as the micro-inertia characteristic length (the scaling parameter for dynamics), and the dot over the vector component $u_{i}$ refers to the time derivative. 

The equations of motion can be derived by using variational principles [40,41,42]. Bearing in mind the variations of internal energy δU and kinetic energy δK

$$\delta U=\int_{V}\left(\sigma_{ij}\delta u_{i,j}+\mu_{ijk}\delta u_{i,jk}+D_{k}\delta \varphi_{e,k}\right)dV,\;\;\delta K=\int_{V}\rho \left({\dot{u}}_{i}\delta {\dot{u}}_{i}+l_{1}^{2}{\dot{u}}_{i,j}\delta {\dot{u}}_{i,j}\right)dV$$

in the Hamilton principle

$$\delta \int_{0}^{t}\left(U-W-K\right)d\tau =0$$

with assuming no body sources for the work of external loading δW, the governing equations (i.e., the equation of motion and the Gauss law) become (see [39,43] for more details)

(6) $$\sigma_{ij,j}-\mu_{ijk,jk}=\rho \left(1-l_{1}^{2}\nabla^{2}\right){\ddot{u}}_{i},$$

(7) $$D_{k,k}=0,$$

where ∇ is nabla operator, and the media without free electric charges are considered. Note that focusing on micro-inertia effect in the present study, the employed simplified model does not involve the micro-stiffness effects represented in the density of internal energy by coupling terms $\varepsilon_{ij,k}\varepsilon_{lm,n}$ and $\varepsilon_{ij}\varepsilon_{kl,m}$ [44].

Field Equations (6) and (7), constitutive and kinematic relations (1)–(5) form a complete set of equations of linear strain gradient theory of flexoelectric continua with micro-inertia effect.

### 2.2. Flexoelectric Layer (Domain −h<x<0)

Love waves belong to anti-plane problems. Assuming translational symmetry with respect to *z* coordinate, all field variables are dependent on the *x* and *y* coordinates and time variable *t*. The mechanical displacement, electric field vector, and electric potential are as follows:

$$u=\left(0,0,u_{3}(x,y,t\right),\;\;E=\left(E_{1}(x,y,t),E_{2}(x,y,t),0\right),\;\;\varphi_{e}=\varphi_{e}\left(x,y,t\right).$$

Above nonzero physical variables are substituted into the kinetic relations (4) and (5):

(8) $$\varepsilon_{31}=\frac{1}{2}\frac{\partial u_{3}}{\partial x},\;\;\;\;\varepsilon_{32}=\frac{1}{2}\frac{\partial u_{3}}{\partial y},$$

(9) $$\eta_{131}=\eta_{311}=\frac{1}{2}\frac{\partial^{2}u_{3}}{\partial x^{2}},\;\;\;\;\eta_{232}=\eta_{322}=\frac{1}{2}\frac{\partial^{2}u_{3}}{\partial y^{2}},$$

(10) $$\eta_{231}=\eta_{321}=\eta_{132}=\eta_{312}=\frac{1}{2}\frac{\partial^{2}u_{3}}{\partial x\partial y},$$

(11) $$E_{1}=-\frac{\partial \varphi_{e}}{\partial x},\;\;\;\;E_{2}=-\frac{\partial \varphi_{e}}{\partial y}.$$

Governing Equations (6) and (7) can be written as:

(12) $$\frac{\partial \sigma_{31}}{\partial x}+\frac{\partial \sigma_{32}}{\partial y}-\frac{\partial^{2}\mu_{311}}{\partial x^{2}}-\frac{\partial^{2}\mu_{312}}{\partial x\partial y}-\frac{\partial^{2}\mu_{321}}{\partial x\partial y}-\frac{\partial^{2}\mu_{322}}{\partial y^{2}}=\rho \left[\frac{\partial u_{3}}{\partial t^{2}}-l_{1}^{2}\left(\frac{\partial^{4}u_{3}}{\partial t^{2}\partial x^{2}}+\frac{\partial^{4}u_{3}}{\partial t^{2}\partial y^{2}}\right)\right],$$

(13) $$\frac{\partial D_{1}}{\partial x}+\frac{\partial D_{2}}{\partial y}=0.$$

The constitutive Equations (1)–(3) can be rewritten into the following form

(14) $$\sigma_{31}=2c_{44}\varepsilon_{31}-e_{15}E_{1},$$

(15) $$\sigma_{32}=2c_{44}\varepsilon_{32}-e_{15}E_{2},$$

(16) $$\mu_{311}=-f_{52}E_{2},$$

(17) $$\mu_{321}=-f_{41}E_{1},$$

(18) $$\mu_{312}=-f_{52}E_{1},$$

(19) $$\mu_{322}=f_{41}E_{2},$$

(20) $$D_{1}=a_{11}E_{1}+2e_{15}\varepsilon_{31}+2\left(f_{41}+f_{52}\right)\eta_{321},$$

(21) $$D_{2}=a_{11}E_{2}+2e_{15}\varepsilon_{32}-2f_{52}\eta_{311}-2f_{41}\eta_{322}.$$

Yang et al. [26] notation is adopted here, $f_{2311}=f_{2131}=-f_{52}$, $f_{1312}=f_{1132}=f_{52}$, $f_{1231}=f_{1321}=f_{41}$, $f_{2232}=f_{2322}=-f_{41}$.

Finally, utilizing Equations (8)–(21) the governing equations for the piezoelectric layer can be expressed as

(22) $$c_{44}\left(\frac{\partial^{2}u_{3}}{\partial x^{2}}+\frac{\partial^{2}u_{3}}{\partial y^{2}}\right)+e_{15}\left(\frac{\partial^{2}\varphi_{e}}{\partial y^{2}}+\frac{\partial^{2}\varphi_{e}}{\partial x^{2}}\right)+f_{41}\left(\frac{\partial^{3}\varphi_{e}}{\partial y^{3}}-\frac{\partial^{3}\varphi_{e}}{\partial x^{2}\partial y}\right)=\rho \left[\frac{\partial^{2}u_{3}}{\partial t^{2}}-l_{1}^{2}\left(\frac{\partial^{4}u_{3}}{\partial t^{2}\partial x^{2}}+\frac{\partial^{4}u_{3}}{\partial t^{2}\partial y^{2}}\right)\right],$$

(23) $$-a_{11}\left(\frac{\partial^{2}\varphi_{e}}{\partial x^{2}}+\frac{\partial^{2}\varphi_{e}}{\partial y^{2}}\right)+e_{15}\left(\frac{\partial^{2}u_{3}}{\partial x^{2}}+\frac{\partial^{2}u_{3}}{\partial y^{2}}\right)+f_{41}\left(\frac{\partial^{3}u_{3}}{\partial x^{2}\partial y}-\frac{\partial^{3}u_{3}}{\partial y^{3}}\right)=0.$$

Compared to the classical piezoelectricity, additional terms proportional to the flexoelectric coefficient $f_{41}$ and the micro-inertia characteristic length $l_{1}$ appeared in the system of governing Equations (22) and (23).

Next, a solution to Equations (22) and (23) in the form of plane harmonic wave is assumed. For Love wave propagation in the *y*-direction, the general solution to the mentioned set of equations can be obtained as follows:

(24) $$u_{3}\left(x,y,t\right)=u(x)e^{ik(y-ct)},$$

(25) $$\varphi_{e}\left(x,y,t\right)=\varphi (x)e^{ik(y-ct)},$$

where u(x) and φ(x) are the unknown functions which represent the amplitudes of the mechanical displacement and electrical potential in the layer, *k* is the wave number, *c* is the phase velocity of the Love waves, and *i* is an imaginary unit defined by formula $i=\sqrt{-1}$.

Substitution of relations (24) and (25) into the governing Equations (22) and (23) leads to the set of ordinary differential equations

(26) $$\left(c_{44}-l_{1}^{2}\rho k^{2}c^{2}\right)\frac{d^{2}u}{dx^{2}}+\left[\rho c^{2}\left(1+l_{1}^{2}k^{2}\right)-c_{44}\right]k^{2}u+\left(e_{15}-if_{41}k\right)\frac{d^{2}\varphi }{dx^{2}}-\left(e_{15}+if_{41}k\right)k^{2}\varphi =0,$$

(27) $$a_{11}\left(\frac{d^{2}\varphi }{dx^{2}}-k^{2}\varphi \right)-\left(e_{15}+ikf_{41}\right)\frac{d^{2}u}{dx^{2}}+\left(e_{15}-ikf_{41}\right)k^{2}u=0.$$

It should be noted that if flexoelectricity and micro-inertia effect are excluded, the governing Equations (26) and (27) are reduced to that of classical piezoelectricity. After some mathematical manipulations, Equation (27) can be written as follows:

(28) $$\left[c_{p}^{2}-l_{1}^{2}k^{2}c^{2}+k^{2}d_{f}^{2}\right]\frac{d^{4}\varphi }{dx^{4}}+\left[c^{2}\left(1+2l_{1}^{2}k^{2}\right)-2c_{p}^{2}+2kd_{f}^{2}\right]k^{2}\frac{d^{2}\varphi }{dx^{2}}-\left[c^{2}\left(1+l_{1}^{2}k^{2}\right)-c_{p}^{2}-k^{2}d_{f}^{2}\right]k^{4}\varphi =0.$$

Here, $d_{f}^{2}=f_{41}^{2}/\rho a_{11}$, and $c_{p}=\sqrt{{\bar{c}}_{44}/\rho }$ is the velocity of the shear wave in elastic piezoelectricity, where ${\bar{c}}_{44}=c_{44}+e_{15}^{2}/a_{11}$ is a piezoelectric stiffened elastic constant. 

We represent a solution to Equation (28) in the following form $\varphi ∼B_{\varphi }e^{\lambda x}$. From Equation (28) in order to find the unknown quantity λ, we get biquadratic equation

(29) $$\left[c_{p}^{2}-l_{1}^{2}k^{2}c^{2}+k^{2}d_{f}^{2}\right]\lambda^{4}+\left[c^{2}\left(1+2l_{1}^{2}k^{2}\right)-2c_{p}^{2}+2kd_{f}^{2}\right]k^{2}\lambda^{2}-\left[c^{2}\left(1+l_{1}^{2}k^{2}\right)-c_{p}^{2}-k^{2}d_{f}^{2}\right]k^{4}=0.$$

The solutions to this equation are: $\lambda_{1}=k\Lambda_{1}$, $\lambda_{2}=-k\Lambda_{1}$, $\lambda_{3}=k\Lambda_{2}$, and $\lambda_{4}=-k\Lambda_{2}$, where

(30) $$\Lambda_{1}=\sqrt{\frac{2c_{p}^{2}-c^{2}\left(1+2l_{1}^{2}k^{2}\right)-2k^{2}d_{f}^{2}+\sqrt{D}}{2\left(c_{p}^{2}-l_{1}^{2}k^{2}c^{2}+k^{2}d_{f}^{2}\right)}},$$

(31) $$\Lambda_{2}=\sqrt{\frac{2c_{p}^{2}-c^{2}\left(1+2l_{1}^{2}k^{2}\right)-2k^{2}d_{f}^{2}-\sqrt{D}}{2\left(c_{p}^{2}-l_{1}^{2}k^{2}c^{2}+k^{2}d_{f}^{2}\right)}},$$

(32) $$D=c^{4}+8c^{2}k^{2}d_{f}^{2}\left(1+2l_{1}^{2}k^{2}\right)-16c_{p}^{2}d_{f}^{2}k^{2}.$$

Thus, the general solution to Equations (28) and (26) can be written as follows:

(33) $$u\left(x\right)=GB_{1}e^{k\Lambda_{1}x}+GB_{2}e^{-k\Lambda_{1}x}+QB_{3}e^{k\Lambda_{2}x}+QB_{4}e^{-k\Lambda_{2}x},$$

(34) $$\varphi \left(x\right)=B_{1}e^{k\Lambda_{1}x}+B_{2}e^{-k\Lambda_{1}x}+B_{3}e^{k\Lambda_{2}x}+B_{4}e^{-k\Lambda_{2}x},$$

where

(35) $$G=-\frac{e_{15}\left(\Lambda_{1}^{2}-1\right)-if_{41}k\left(\Lambda_{1}^{2}+1\right)}{\left(c_{44}-l_{1}^{2}\rho c^{2}k^{2}\right)\left(\Lambda_{1}^{2}-1\right)+\rho c^{2}},$$

(36) $$Q=-\frac{e_{15}\left(\Lambda_{2}^{2}-1\right)-if_{41}k\left(\Lambda_{2}^{2}+1\right)}{\left(c_{44}-l_{1}^{2}\rho k^{2}c^{2}\right)\left(\Lambda_{2}^{2}-1\right)+\rho c^{2}}.$$

Here, $B_{1},B_{2},B_{3}$, and $B_{4}$ are unknown constants to be determined. 

Recall that the applied model does not incorporate the micro-stiffness effects occurring in the higher-grade theory of elasticity and plays a role in size-effects observed in micro/nano size samples. If the micro-stiffness terms are considered in the higher-order stress tensor (2), the governing fourth order differential Equation (28) is replaced by the sixth order equation and thus the dispersion equation becomes very complicated. However, the aim of the authors is to extend the study of propagation of Love waves in thin layers with also including the micro-stiffness effects in future works.

### 2.3. The Air (Domain x<−h)

Since the layer is a piezoelectric medium, we take the electric field in the domain x<−h into account. The air can be regarded as a vacuum and the electric potential in the vacuum is determined by the Laplace equation $\nabla^{2}\varphi_{e}^{v}=0$. Here, $\nabla^{2}$ is the two-dimensional Laplacian operator, and superscript ‘*v*’ indicates the electric potential in domain x<−h. The electric potential in the air tends to zero far away from the surface x=−h along the negative *x*-direction, that is, $\varphi_{e}^{v}\to 0$ as x→−∞. Furthermore, the electric potential is to be continuous on the interface x=−h. Therefore, the electric field above the layer is given by the expression:

(37) $$\varphi_{e}^{v}\left(x,y,t\right)=B_{0}e^{kx}e^{ik(y-ct)},$$

where $B_{0}$ is the unknown constant. The electric displacement in the air is as follows:

(38) $$D_{1}^{v}=-a_{0}\frac{\partial \varphi_{e}^{v}}{\partial x}=-kB_{0}a_{0}e^{kx}e^{ik(y-ct)},\;\;\;D_{2}^{v}=-a_{0}\frac{\partial \varphi_{e}^{v}}{\partial y}=-ikB_{0}a_{0}e^{kx}e^{ik(y-ct)},\;\;\mathrm{at}\;x<-h.$$

Here, $a_{0}$ is the dielectric constant of the air (vacuum). 

### 2.4. Piezoelectric Substrate (Domain x>0)

The substrate is considered as a piezoelectric elastic material. Because of huge dimensions of the substrate, the flexoelectricity and micro-inertia effects are supposed to be negligible. All quantities related to half-space will be identified by superscript *h,* namely, the material coefficients of the substrate are given by $\rho^{h}$, $c_{ijkl}^{h}$, $e_{kij}^{h}$, $a_{ij}^{h}$. Quantities that characterize the wave propagation within the piezoelectric substrate (domain x>0) are:

$$u^{h}=\left(0,0,u_{3}^{h}(x,y,t\right),\;\;\;\;E^{h}=\left(E_{1}^{h}(x,y,t),E_{2}^{h}(x,y,t),0\right),\;\;\varphi_{e}^{h}=\varphi_{e}^{h}\left(x,y,t\right).$$

(39) $$\varepsilon_{13}^{h}=\frac{1}{2}\frac{\partial u_{3}^{h}}{\partial x},\;\;\;\varepsilon_{32}^{h}=\frac{1}{2}\frac{\partial u_{3}^{h}}{\partial y},\;\;\;E_{1}^{h}=-\frac{\partial \varphi_{e}^{h}}{\partial x},\;\;\;E_{2}^{h}=-\frac{\partial \varphi_{e}^{h}}{\partial y}.$$

Bearing in mind the continuity requirements on the interface x=0, the displacement component and the electric potential are assumed as

(40) $$u_{3}^{h}\left(x,y,t\right)=u^{h}(x)e^{ik(y-ct)},$$

(41) $$\varphi_{e}^{h}\left(x,y,t\right)=\varphi^{h}(x)e^{ik(y-ct)}.$$

Here, $u^{h}(x)$ and $\varphi^{h}(x)$ are the unknown functions which represent the amplitudes of the mechanical displacement and electrical potential in half-space.

Within the linear piezoelectricity, the constitutive relations can be written as:

(42) $$\sigma_{31}^{h}=2c_{44}^{h}\varepsilon_{31}^{h}-e_{15}^{h}E_{1}^{h},$$

(43) $$\sigma_{32}^{h}=2c_{44}^{h}\varepsilon_{32}^{h}-e_{15}^{h}E_{2}^{h},$$

(44) $$D_{1}^{h}=a_{11}^{h}E_{1}^{h}+2e_{15}^{h}\varepsilon_{13}^{h},$$

(45) $$D_{2}^{h}=a_{11}^{h}E_{2}^{h}+2e_{15}^{h}\varepsilon_{32}^{h}.$$

For deformable piezoelectric half-space without micro-inertia terms, the governing set of differential equations reduces to:

(46) $$c_{44}^{h}\frac{d^{2}u^{h}}{dx^{2}}-\left(c_{44}^{h}-\rho^{h}c^{2}\right)k^{2}u^{h}+e_{15}^{h}\left(\frac{d^{2}\varphi^{h}}{dx^{2}}-k^{2}\varphi^{h}\right)=0,$$

(47) $$\left(\frac{d^{2}\varphi^{h}}{dx^{2}}-k^{2}\varphi^{h}\right)=\frac{e_{15}^{h}}{a_{11}^{h}}\left(\frac{d^{2}u^{h}}{dx^{2}}-k^{2}u^{h}\right).$$

Excluding the electric field components from Equation (46), we get ordinary differential equation for the displacement

(48) $${\bar{c}}_{44}^{h}\frac{d^{2}u^{h}}{dx^{2}}-\left({\bar{c}}_{44}^{h}-\rho^{h}c^{2}\right)k^{2}u^{h}=0,$$

where ${\bar{c}}_{44}^{h}=c_{44}^{h}\left(1+\frac{{\left(e_{15}^{h}\right)}^{2}}{c_{44}^{h}a_{11}^{h}}\right)$ is a piezoelectric stiffened elastic constant, and $\kappa =\frac{{\left(e_{15}^{h}\right)}^{2}}{c_{44}^{h}a_{11}^{h}}$ is the dimensionless number called the electromechanical coupling factor.

Since the displacement and electric potential in the substrate should tend to zero far away from the interface (that is, $u_{3}^{h}\to 0,\;\varphi_{e}^{h}\to 0$ as x→+∞) a general solution for Equations (47) and (48) is given as:

(49) $$u_{3}^{h}\left(x,y,t\right)=C_{1}e^{-\beta kx}e^{ik(y-ct)},$$

(50) $$\varphi_{e}^{h}\left(x,y,t\right)=\left(\frac{e_{15}^{h}}{a_{11}^{h}}C_{1}e^{-\beta kx}+C_{2}e^{-kx}\right)e^{ik(y-ct)}.$$

Here, $C_{1}$, $C_{2}$ are constants, $\beta =\sqrt{1-c^{2}/{\left(c_{p}^{h}\right)}^{2}}$, and $c_{p}^{h}=\sqrt{{\bar{c}}_{44}^{h}/\rho^{h}}$ is the velocity of the shear wave in elastic piezoelectric substrate. From the above formula we conclude that the velocity of the Love waves propagating in dielectric material must satisfy the condition $c<c_{p}^{h}$.

### 2.5. Boundary Conditions

The unknown constants $B_{0}$, $B_{1},B_{2},B_{3}$, $B_{4}$, $C_{1}$, and $C_{2}$ should be determined by the boundary conditions at surfaces x=−h and x=0. The generalized tractions on the boundary of a subdomain in plane *x-y* are given as $t_{i}=\left(\sigma_{ij}-\mu_{ijk,k}+\rho l_{1}^{2}{\ddot{u}}_{i,j}\right)n_{j}-n_{k}\tau_{j}\mu_{ijk,l}\tau_{l}$, where $n_{j}$ ($\tau_{j}$) are components of the unit normal (tangent) vector on the considered boundary contour. The surface x=−h is free of tractions while the displacement and tangential stress components are continuous on the interface boundary x=0. At the interface between the layer and the substrate (x=0), both the electric potential and the normal component of electric displacements are continuous. Either the electric open- or short-circuit conditions are realized at the layer upper surface x=−h.

(I) In case of the electric open-circuit conditions, we require the flux of electric displacements and electric potentials to be continuous across the surface x=−h. Thus, we consider the complete boundary conditions as:

On the surface x=−h:

The traction boundary condition:

(51) $${\left.\left(\left(\sigma_{31}-\mu_{311,1}-\mu_{312,2}\right)-\mu_{321,2}+\rho l_{1}^{2}\frac{\partial {\ddot{u}}_{3}}{\partial x}\right)\right|}_{x=-h}=0,$$

The electric open-circuit conditions:

(52) $${\left.\varphi_{e}\right|}_{x=-h}={\left.\varphi_{e}^{v}\right|}_{x=-h},\;\;\;{\left.D_{1}\right|}_{x=-h}={\left.D_{1}^{v}\right|}_{x=-h}.$$

On the interface between the layer and half-space. x=0:

The mechanical boundary conditions:

(53) $${\left.u_{3}\right|}_{x=0}={\left.u_{3}^{h}\right|}_{x=0},$$

(54) $${\left.\left(\left(\sigma_{31}-\mu_{311,1}-\mu_{312,2}\right)-\mu_{321,2}+\rho l_{1}^{2}\frac{\partial {\ddot{u}}_{3}}{\partial x}\right)\right|}_{x=0}={\left.\sigma_{31}^{h}\right|}_{x=0},$$

The electric boundary conditions:

(55) $${\left.\varphi_{e}\right|}_{x=0}={\left.\varphi_{e}^{h}\right|}_{x=0},\;\;\;\;{\left.D_{1}\right|}_{x=0}={\left.D_{1}^{h}\right|}_{x=0}.$$

Here, $\tau_{31}=\left(\sigma_{31}-\mu_{311,1}-\mu_{312,2}\right)-\mu_{321,2}+\rho l_{1}^{2}\frac{\partial {\ddot{u}}_{3}}{\partial x}$ is the *z*-component of generalized tractions on the surfaces $\left(x=const,y,z\right)$, $y,z\in \left(-\infty ,\infty \right)$.

(II) Under the electric short-circuit condition, the electric potential at the layer surface x=−h is equal to zero. In this case, Equation (52) should be replaced by the following relation:

(56) $${\left.\varphi_{e}\right|}_{x=-h}=0.$$

Firstly, let us consider the electric open-circuit condition. Substitution of the general solutions (33), (34), (37), (38), (49) and (50) into mechanical and electric boundary conditions (51)–(55) produces seven homogeneous algebraic linear equations to find unknown constants $B_{0}$, $B_{1},B_{2},B_{3}$, $B_{4}$, $C_{1}$, and $C_{2}$. Eliminating $B_{0}$, $C_{1}$, and $C_{2}$ from the obtained set of equations we obtain four equations with respect to *B*_1_, *B*_2_, *B*_3_, and *B*_4_ which can be written in a matrix form as follows: MB=0, where $B^{T}=\left[\begin{array}{cccc} B_{1} & B_{2} & B_{3} & B_{4} \end{array}\right]$, and the matrix **M** looks as:

$$M=\left[\begin{array}{cccc} m_{11}e^{-\Lambda_{1}kh} & -m_{11}e^{\Lambda_{1}kh} & m_{12}e^{-\Lambda_{2}kh} & -m_{12}e^{\Lambda_{2}kh}\\ -m_{21}e^{-\Lambda_{1}kh} & m_{21}e^{\Lambda_{1}kh} & -m_{22}e^{-\Lambda_{2}kh} & m_{22}e^{\Lambda_{2}kh}\\ \left(m_{32}+m_{31}\right) & \left(m_{32}-m_{31}\right) & \left(m_{34}+m_{33}\right) & \left(m_{34}-m_{33}\right)\\ \left(m_{42}-m_{41}\right) & \left(m_{42}+m_{41}\right) & \left(m_{44}-m_{43}\right) & \left(m_{44}+m_{43}\right) \end{array}\right].$$

The elements of matrix **M** are given by formulae:

$$\begin{array}{c} m_{11}=\left(c_{44}G-\rho l_{1}^{2}k^{2}c^{2}G+e_{15}-ikf_{41}\right)\Lambda_{1},\\ m_{12}=\left(c_{44}Q-\rho l_{1}^{2}k^{2}c^{2}Q+e_{15}-ikf_{41}\right)\Lambda_{2},\\ m_{21}=\left[a_{11}-e_{15}G-ikG\left(f_{41}+f_{52}\right)\right]\Lambda_{1}+a_{0},\\ m_{22}=\left[a_{11}-e_{15}Q-ikQ\left(f_{41}+f_{52}\right)\right]\Lambda_{2}+a_{0},\\ m_{31}=\left[G\left(c_{44}+\frac{e_{15}e_{15}^{h}}{a_{11}^{h}}-\rho l_{1}^{2}k^{2}c^{2}\right)+e_{15}-e_{15}^{h}\frac{a_{11}}{a_{11}^{h}}+ik\left(\left(f_{41}+f_{52}\right)\frac{e_{15}^{h}}{a_{11}^{h}}G-f_{41}\right)\right]\Lambda_{1},\\ m_{32}=G{\bar{c}}_{44}^{h}\beta ,\\ m_{33}=\left[Q\left(c_{44}+\frac{e_{15}e_{15}^{h}}{a_{11}^{h}}-\rho l_{1}^{2}k^{2}c^{2}\right)+e_{15}-e_{15}^{h}\frac{a_{11}}{a_{11}^{h}}+ik\left(\left(f_{41}+f_{52}\right)\frac{e_{15}^{h}}{a_{11}^{h}}Q-f_{41}\right)\right]\Lambda_{2},\\ m_{34}=Q{\bar{c}}_{44}^{h}\beta ,\\ m_{41}=\left[a_{11}-e_{15}G-ikG\left(f_{41}+f_{52}\right)\right]\Lambda_{1},\\ m_{42}=Ge_{15}^{h}-a_{11}^{h},\\ m_{43}=\left[a_{11}-e_{15}Q-ikQ\left(f_{41}+f_{52}\right)\right]\Lambda_{2},\\ m_{44}=Qe_{15}^{h}-a_{11}^{h}. \end{array}$$

From the above equations one can observe that the piezoelectricity-related terms are independent of the wave number *k*, the flexoelectricity-related terms are dependent on *k*, while the micro-inertia-related terms are dependent on $k^{2}$.

Under the electric short-circuit condition, in order to find unknown constants, Equations (51), (53)–(56) should be used. For this case, matrix **M** looks as follows:

$$M=\left[\begin{array}{cccc} m_{11}e^{-\Lambda_{1}kh} & -m_{11}e^{\Lambda_{1}kh} & m_{12}e^{-\Lambda_{2}kh} & -m_{12}e^{\Lambda_{2}kh}\\ e^{-\Lambda_{1}kh} & e^{\Lambda_{1}kh} & e^{-\Lambda_{2}kh} & e^{\Lambda_{2}kh}\\ \left(m_{32}+m_{31}\right) & \left(m_{32}-m_{31}\right) & \left(m_{34}+m_{33}\right) & \left(m_{34}-m_{33}\right)\\ \left(m_{42}-m_{41}\right) & \left(m_{42}+m_{41}\right) & \left(m_{44}-m_{43}\right) & \left(m_{44}+m_{43}\right) \end{array}\right].$$

To obtain nontrivial solutions for B, the determinant of matrix **M** should vanish. This leads to the dispersion equation

(57) $$\det\left[M(c,k)\right]=0.$$

This dispersion relation determines the dependence of the Love-wave phase velocity *c* on the wave numbers *k*, i.e., *c* = *c*(*k*). Due to the presence of both the flexoelectricity and micro-inertia effects in the piezoelectric layer, the dispersion relation (57) becomes very complicated and numerical methods should be applied to solve it.

## 3. Numerical Solution and Results

Numerical results devoted to the phase velocity versus wave number are presented in this paragraph for both the electric open- and short-circuit conditions on the layer-vacuum interface. The roots of the dispersion relation (57) yield a series of modes of wave propagation. Since the first mode is characterized by the largest amplitude, we analyze the behavior of this mode only. Following [26], we assume the wave number to be positive, real quantity while the Love wave phase velocity *c* is considered as a complex one, i.e., $c=c_{1}+ic_{2}$. The imaginary part of velocity $c_{2}$ characterizes the surface wave attenuation. The negative values of $c_{2}$ mean that the wave amplitude drops, and vice versa the positive values of $c_{2}$ mean that the wave amplitude grows.

Barium titanate and lithium niobate were considered as materials for the piezoelectric half-space in numerical analyses. The layer material should have a slower shear bulk velocity than the substrate. Hence, we considered the lead zirconate titanate as flexoelectric material for the layer. Thereby, we consider the structure with PZT-5H layer deposited on the BaTiO_3_ or LiNbO_3_ substrate. These material combinations satisfy the above-mentioned condition for the wave propagation. The material coefficients used in the calculation are provided in Table 1.

In the vacuum, the dielectric constant is $a_{0}=8.85\times 10^{-12}F/m$. The thickness *h* of the flexoelectric layer is taken from the range of nanometers, when the nano-scale effects play a role. The micro-inertia length $l_{1}$ is a characteristic of the microstructure of the material. The dynamic characteristic length $l_{1}$ is set to be proportional to the lattice parameter *a*, which is equal to 4Ǻ for PZT-5H [47]. Following [33], we assume $l_{1}$ to range from the lattice parameter *a* to several times of its value in the presented parametric study.

**Figure 2:**
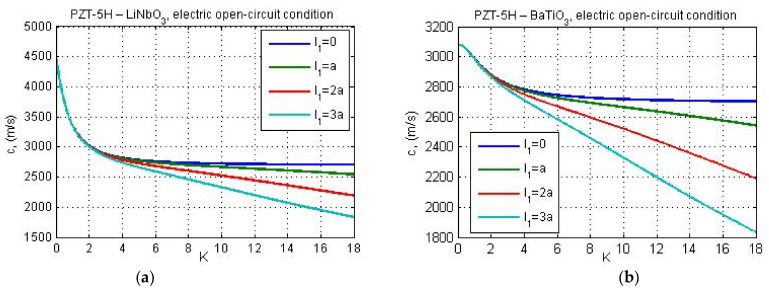
Phase velocity of Love wave versus non-dimensional wave number *K* = *kh* for two substrate materials: (**a**) LiNbO_3_, and (**b**) BaTiO_3_ and for different micro-inertia characteristic lengths in case of electric open circuit conditions.

**Figure 3:**
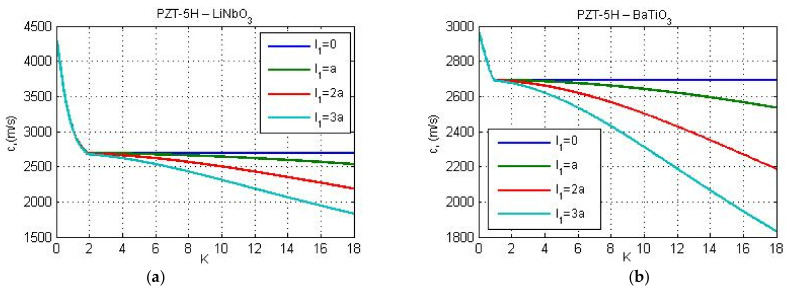
Phase velocity of Love wave versus non-dimensional wave number *K = kh* for two substrate materials: (**a**) LiNbO_3_, and (**b**) BaTiO_3_ and for different micro-inertia characteristic lengths in case of electric short circuit conditions.

**Figure 4:**
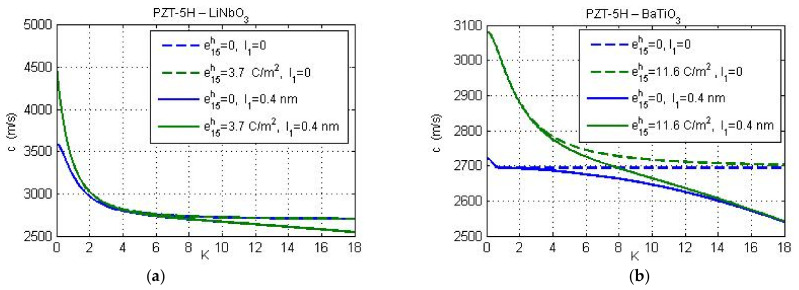
Influence of the piezoelectricity on the phase velocity *c* for two substrate materials: (**a**) LiNbO_3_, and (**b**) BaTiO_3_.

In Figure 2, Figure 3 and Figure 4, the Love wave propagation is calculated by considering the piezoelectricity and micro-inertia effect. Here, flexoelectric properties of the guiding layer are not taken into account. Since flexoelecticity is not considered the elements of matrix **M** are simplified. For this particular case, from Equations (30)–(32), (35) and (36) we get G=0, $Q=a_{11}/e_{15}$, $\Lambda_{1}=1$, and $\Lambda_{2}=i\sqrt{c^{2}/{\tilde{c}}_{p}^{2}-1}$, where, $c_{p}^{}$ is the velocity of the shear wave in classical piezoelectricity, and ${\tilde{c}}_{p}=\sqrt{c_{p}^{2}-l_{1}^{2}k^{2}c^{2}}$ is the velocity of the shear wave in piezoelectric continuum with micro-inertia effect. However, despite some simplifications of the matrix **M**, the dispersion Equation (57) still remains very complicated. Therefore, the phase velocity is computed numerically from dispersion relation (57) using MATLAB.

Figure 2 and Figure 3 illustrate the influence of micro-inertia parameter on the phase velocity of Love wave under electric open- and short-circuit conditions, respectively. The material properties of the layer and substrate are chosen as follows: PZT-5H—LiNbO_3_ (Figure 2a and Figure 3a) and PZT-5H—BaTiO_3_ (Figure 2b and Figure 3b). The thickness *h* of the guiding layer is taken to be 20 nm. The line with $l_{1}=0$ corresponds to the classical solution (without flexoelectricity and micro-inertia effects). From Figure 3 it is observed that in the case of electric short circuit conditions within the classical theory, the dispersion curve is horizontal if the wave number reaches some critical value $K_{*}$, where $K_{*}=1.9063$ for PZT-5H—LiNbO_3_ and $K_{*}=0.972$ for PZT-5H—BaTiO_3_. This leads to a constant wave velocity with the increase of wave number ($K>K_{*}$). Note that similar dispersion curve with constant phase velocity within the classical piezoelectricity has been also obtained by Yang and co-authors for a layered structure with material properties ‘LiNbO_3_—Si’ (see Figure 6a in [26]). As seen from Figure 3, when the micro-inertia effect is considered, the velocity of Love waves depends on wave number. In case of electric short circuit condition, the critical value $K_{*}$ determines the range of wave numbers $K>K_{*}$ for which the influence of micro-inertia terms on phase velocity becomes significant. The effect of micro-inertia parameter is more visible for the material combination ‘PZT-5H—BaTiO_3′_. Since flexoelectricity is not taken into account, the phase velocity is real. With increasing the wave number, the phase wave velocity is decreasing in both electric open and short circuit conditions. The reduction is stronger for larger values of the micro-inertia parameter. This conclusion is consistent with the theoretical finding by Polyzos et al. [48] in gradient elasticity models without electro-elastic effects. A similar result was also obtained by Yang with coworkers [33] who studied the micro-inertia effect on the phase velocity of Rayleigh waves. They showed that the micro-inertia term decreases the phase velocity of Rayleigh waves and the influence of mentioned effect is more remarkable for greater wave numbers and micro-inertia characteristic lengths. 

The phase velocity of the Love-wave depends on the material and geometric parameters of the layered structure. The effect of piezoelectric properties of the substrate on the wave velocity *c* is illustrated in Figure 4 for the case of electric-open circuit conditions on the layer-vacuum interface. Again, the guiding layer thickness is 20 nm and the micro-inertia characteristic length is either vanishing or equal to the lattice parameter *a*, i.e., *l*_1_ = *a*. The phase velocity is presented as the function of non-dimensional wave number *K = kh*. The flexoelectric properties of the guiding layer are not considered again. The corresponding classical solutions, $l_{1}=0$, for elastic and piezoelectric substrates are presented by dashed lines there. The increase of piezoelectricity of the substrate increases the phase velocity mainly for smaller wave numbers. The influence of the micro-inertia effect is more remarkable for short wavelengths. For large values of wave number, the phase velocity is smaller than the one predicted by the classical theory. The influence of piezoelectric properties of substrate and the micro-inertia effect is more pronounced for the substrate BaTiO_3_ with PZT-5H layer.

**Figure 5:**
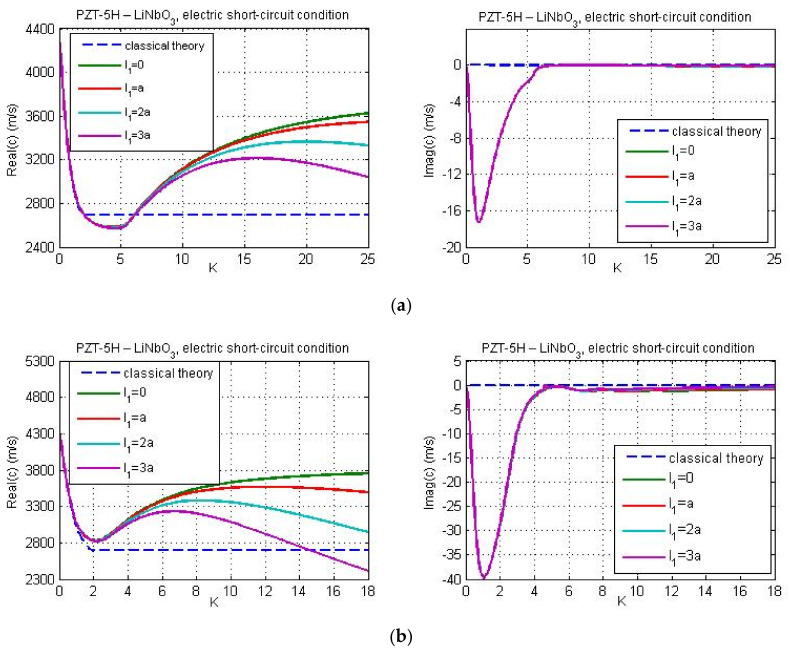
The real and imaginary parts of phase velocity *c* versus non-dimensional wave number *K* for different values of micro-inertia length in case of electric short-circuit conditions; guiding layer thicknesses: (**a**) 60 nm and (**b**) 25 nm.

**Figure 6:**
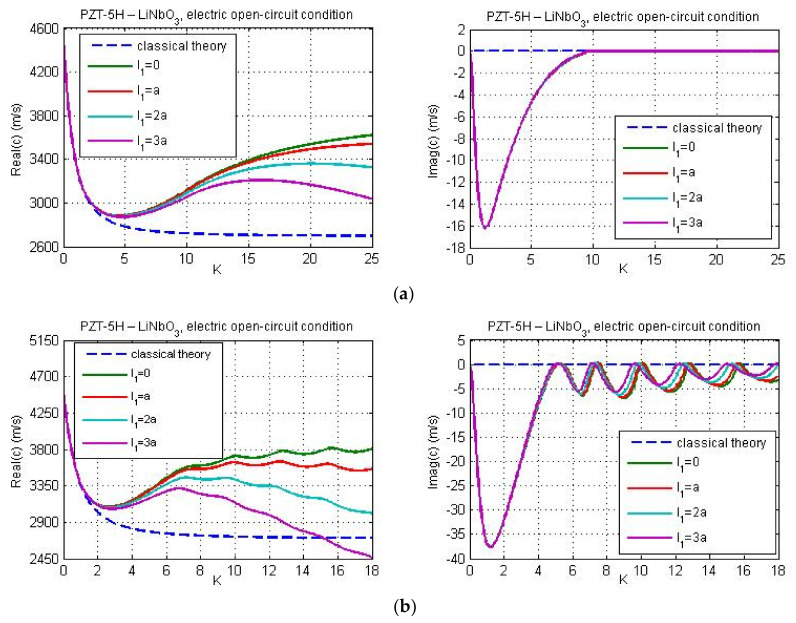
The real and imaginary parts of phase velocity *c* versus non-dimensional wave number *K* for different values of micro-inertia characteristic lengths in case of electric open-circuit conditions; guiding layer thicknesses: (**a**) 60 nm and (**b**) 25 nm.

**Figure 7:**
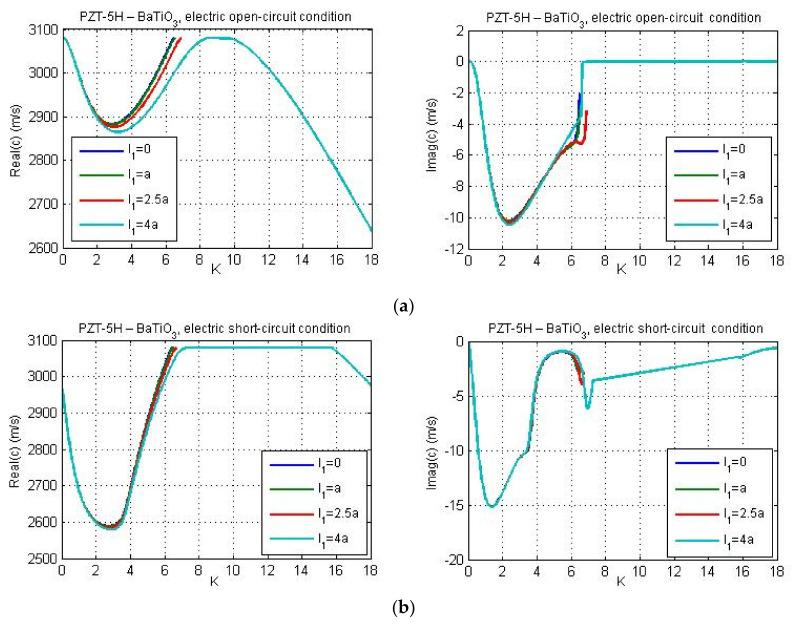
The real and imaginary parts of phase velocity *c* versus non-dimensional wave number *K* for various micro-inertia characteristic lengths; (**a**) electric open-circuit conditions and (**b**) ) electric short-circuit conditions.

**Figure 8:**
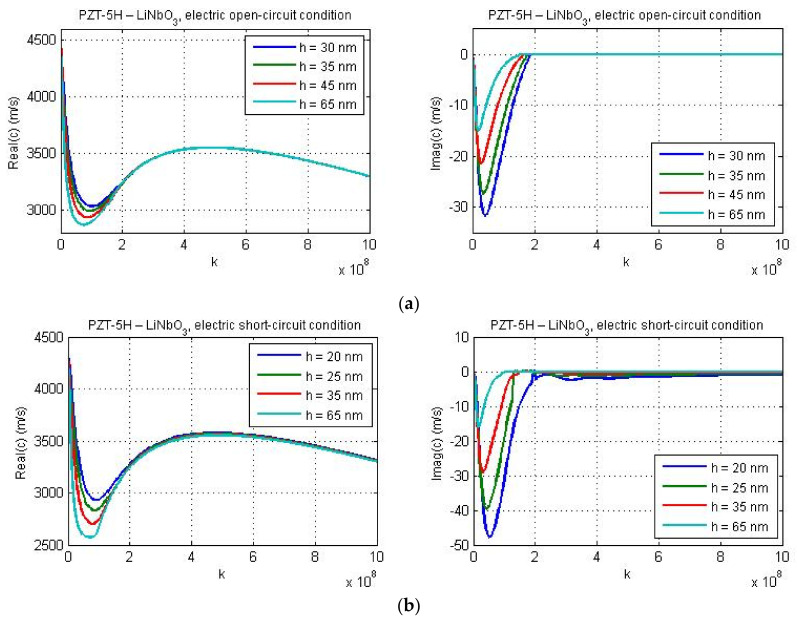
The effect of the guiding layer thickness on the real and imaginary parts of phase velocity for the data of materials PZT-5H—LiNbO_3_; (**a**) electric open-circuit conditions and (**b**) electric short-circuit conditions.

**Figure 9:**
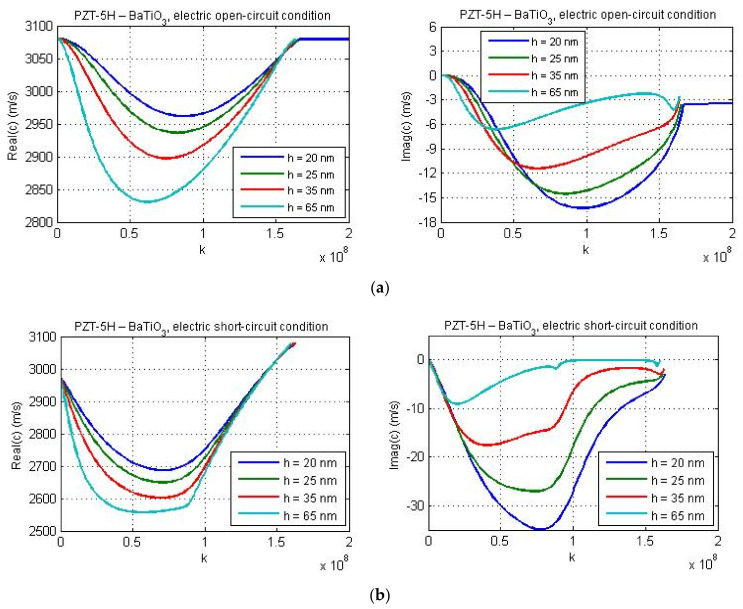
The effect of the guiding layer thickness on the real and imaginary parts of phase velocity for the data of materials PZT-5H—BaTiO_3_; (**a**) electric open-circuit conditions and (**b**) electric short-circuit conditions.

**Figure 10:**
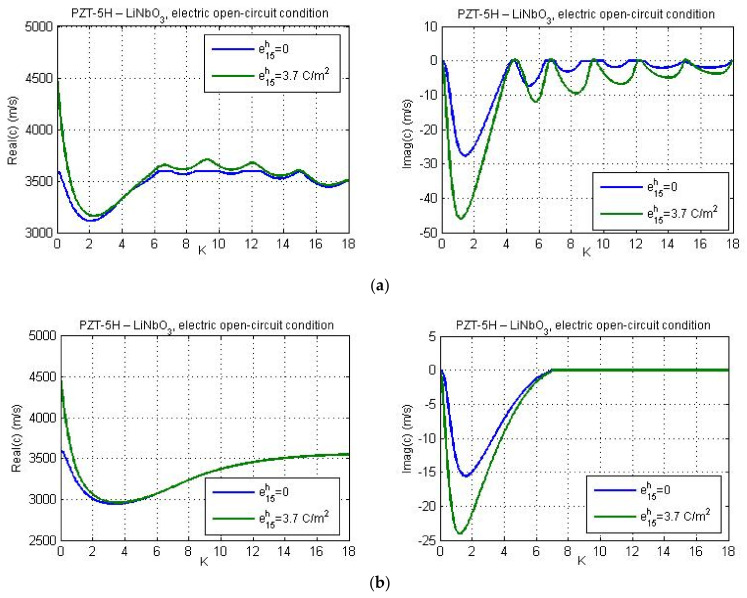
The dispersion curves for the material combination PZT-5H—LiNbO_3_ with and without considering the effect of piezoelectric properties of the substrate under electric open-circuit conditions and for guiding layer thickness: (**a**) *h* = 20 nm and (**b**) *h* = 40 nm.

**Figure 11:**
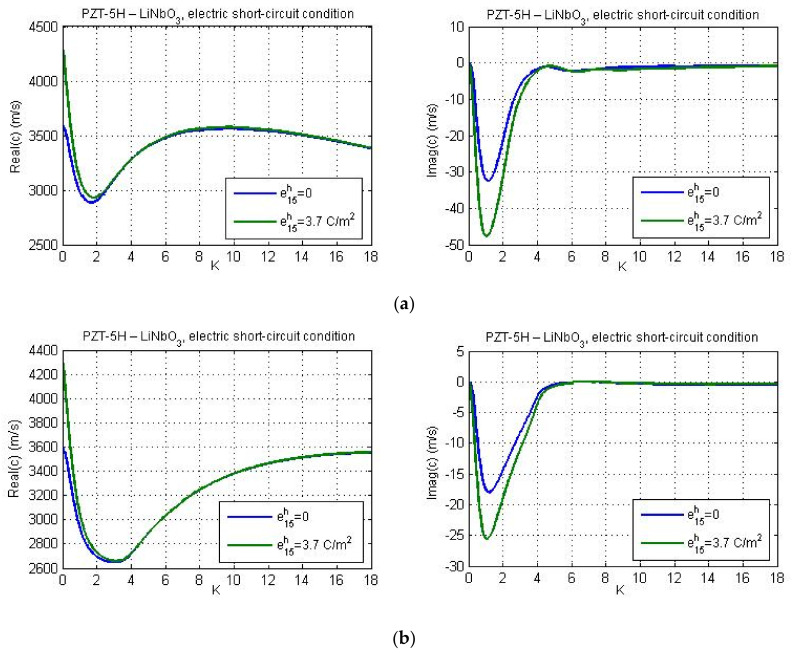
The dispersion curves for the material combination PZT-5H—LiNbO_3_ with and without considering the effect of piezoelectric properties of the substrate under electric short-circuit conditions and for guiding layer thickness: (**a**) *h* = 20 nm and (**b**) *h* = 40 nm.

**Figure 12:**
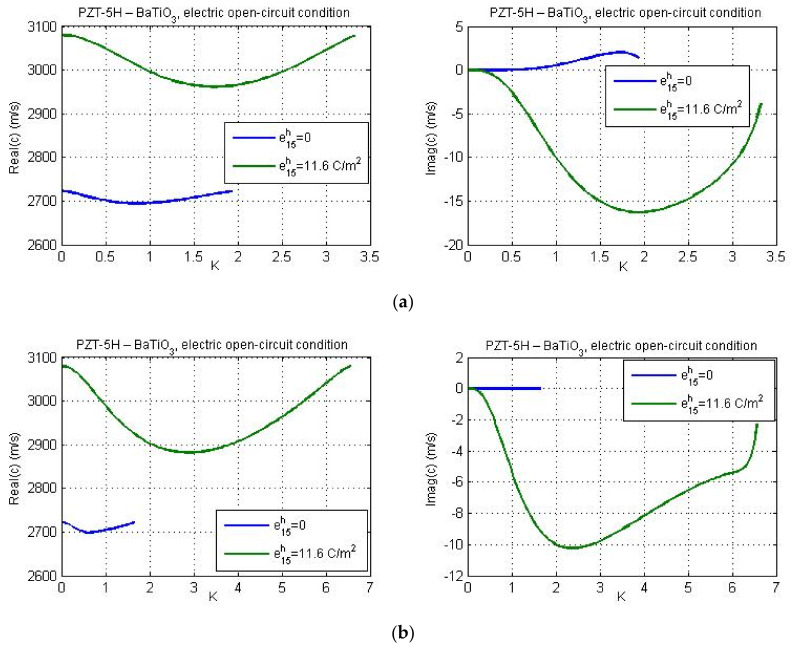
The dispersion curves for the material combination PZT-5H—BaTiO_3_ with and without considering the effect of piezoelectric properties of the substrate under electric open-circuit conditions and for guiding layer thickness: (**a**) *h* = 20 nm and (**b**) *h* = 40 nm.

**Figure 13:**
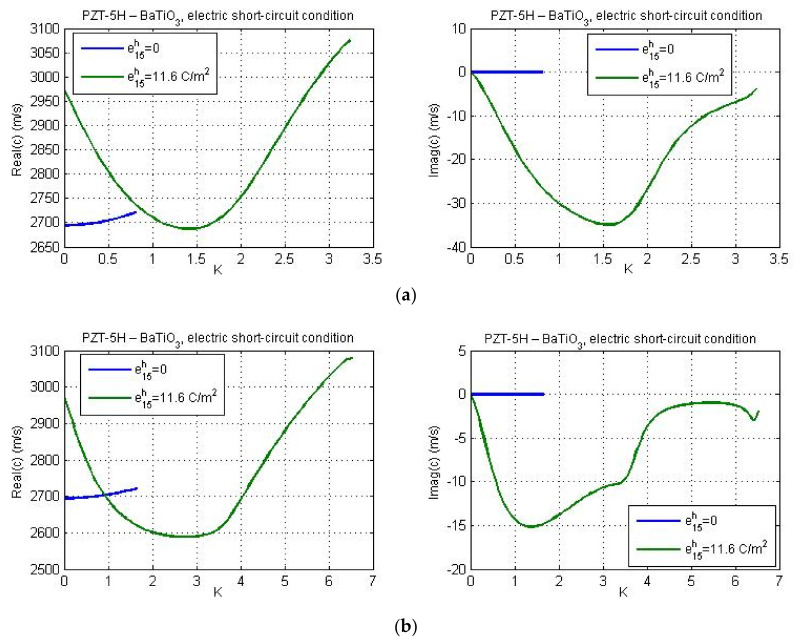
The dispersion curves for the material combination PZT-5H—BaTiO_3_ with and without considering the effect of piezoelectric properties of the substrate under electric short-circuit conditions and for guiding layer thickness: (**a**) *h* = 20 nm and (**b**) *h* = 40 nm.

Results for complex influence of micro-inertia effect, piezoelectric, and flexoelectric properties of the material are presented in Figure 5, Figure 6, Figure 7, Figure 8, Figure 9, Figure 10, Figure 11, Figure 12 and Figure 13. In our calculations, the direct flexoelectricity coefficients $f_{41}$ and $f_{52}$ are considered as $10^{-7}C/m$ [43]. For finite values of flexoelectric parameters the imaginary part of phase velocity *c*_2_ is different from zero, which yields a finite attenuation of the wave amplitude.

Real and imaginary parts of phase velocity are illustrated in Figure 5 and Figure 6 for the LiNbO_3_ substrate material and electric short- and open-circuit conditions, respectively. There are considered four various values for the micro-inertia characteristic length, *l*_1_ = 0; *a*; 2*a*; 3*a*, and two different values of the guiding layer thickness (60 nm and 25 nm). Dashed lines correspond to the classical solutions without flexoelectricity and micro-inertia effect. Comparing results in Figure 3a, Figure 4a, and Figure 5, one can observe a significant influence of flexoelectricity on the profile of the dispersion curves. The real part of phase velocity first decreases, reaches the minimum, and then slowly rises. However, when the dynamic characteristic length is sufficiently large, the real part of the phase velocity reaches some specific value and then it again decreases with an increase in wave number. This effect became more pronounced for the narrower guiding layer (Figure 5b). Increasing micro-inertia parameter, the phase velocity is decreasing. Again, this microstructural effect is more expressive in case of narrower guiding layer.

As shown in Figure 5, the imaginary part of phase velocity first rapidly decreases, reaches its minimum, and then increases with an increment of the wave number and tends to zero. Since the imaginary part of the wave velocity is negative, the wave amplitude attenuates. The minimum of imaginary part of phase velocity corresponds to the maximum wave attenuation. Note that the wave attenuation is more expressive in case of narrower guiding layer.

According to Figure 6a, in case of the electric open-circuit conditions, the profiles of dispersion curves follow the same trends as in Figure 5 if the layer thickness is large. However, when the guiding layer thickness becomes very small the profiles of dispersion curves for large wave numbers change dramatically (see Figure 6b). In this case, both real and imaginary part of phase velocity becomes ‘wavy’ and the Love wave amplitude drops even for large *K*. When the layer thickness is equal to 25 nm the imaginary part of phase velocity can reach positive values for certain wave numbers. It means that for these wave numbers the wave amplitude can grow a bit. A similar result was also obtained by Yang with coworkers [26] who studied the behavior of Love waves in layered flexoelectric structures with material properties ‘LiNbO_3_—Si’. They showed that the presence of flexoelectricity leads to a complex phase velocity with a negative/positive imaginary part.

Figure 7 gives profiles of real and imaginary parts of phase velocity for PZT-5H—BaTiO_3_ materials and guiding layer thickness *h* = 40 nm. The flexoelectricity is considered here. Essential distinctions can be observed between these results and the plots presented in Figure 2b and Figure 3b. Figure 7 demonstrates that the real part of phase velocity first decreases and after reaching the minimum, one observes a rise. If the flexoelectricity is not considered (Figure 2 and Figure 3), the dispersion curves decrease monotonically and the cut-off region is absent. On the other hand, in case of consideration of flexoelectricity, the ‘cut-off wave numbers’ appear for the chosen material combination of the guiding layer and substrate, if the micro-inertia length is finite. The cut-off wave number sets the cut-off wavelength as the maximum wavelength at which the Love wave is capable of propagating. The cut-off region appears in such a case if the real part of phase velocity reaches and exceeds the shear wave velocity in a piezoelectric substrate. From Figure 7 it is observed that in general, the profiles of real and imaginary parts of phase velocity under electric open- and short-circuit conditions display the same trends. There are some differences at large wave numbers and for larger micro-inertia characteristic length. According to Figure 7, when the micro-inertia characteristic length is less than 2.5a, the dispersion curves calculated for different characteristic lengths are close to each other, but if the characteristic length becomes relatively large, the profile of the dispersion curve changes significantly and the cut-off region does not occur, because the influence of micro-inertia terms becomes dominant. In other words, the profile of dispersion curves (including the presence/absence of cut-off regions therein) depends strongly on the relations between the values of flexoelectric coefficients and the micro-inertia characteristic length. It should be noted that the cut-off region for a layered structure with material properties ‘LiNbO_3_—Si’ have been also obtained by Yang and co-authors [26] who studied the effect of flexoelectricity on Love wave propagation without the micro-inertia effect.

The dispersion curves for material combinations ‘PZT-5H—LiNbO_3_′ and ‘PZT-5H—BaTiO_3_′ are presented in Figure 8 and Figure 9 for *l*_1_ = *a* and for various values of the guiding layer thickness.

Figure 8a,b show the dispersion curves for different values of the thickness of PZT-5H layer deposited on the LiNbO_3_ substrate under electric open- and short-circuit conditions, respectively. The effect of layer thickness on Love wave phase velocity is more pronounced for lower wave numbers and at more narrow layers. In both the electrically open and short circuits, the minimum value of real(c) decreases and the minimum for imag(c) increases if the guiding layer thickness increases. Reducing the layer thickness the wave attenuation is enhanced for low *k*.

Figure 9 illustrates the dispersion curves for the material combination PZT-5H—BaTiO_3_ for guiding layer thicknesses 20 nm, 25 nm, 35 nm, and 65 nm. Figure 9a,b present the results for the electric open- and short-circuit conditions, respectively. One can observe a reduction of the wave phase velocity for larger values of the layer thickness in both cases. Since the micro-inertia characteristic length was chosen quite small (*l*_1_ = *a*), for the considered material combination PZT-5H—BaTiO_3_, the cut-off regions are reached for dispersion curves under the electric short-circuit conditions (Figure 9b). The cut-off regions also occur under the electric open-circuit conditions, but only when the layer thickness is equal to 25 nm, 35 nm, and 65 nm. However, the cut-off region is absent if the thickness of guiding layer is equal to 20 nm (see Figure 9a).

The influence of the piezoelectricity of substrate on dispersion curves for the material combination PZT-5H—LiNbO_3_ is presented in Figure 10 and Figure 11 for electric open and short-circuit conditions, respectively. It is observed that piezoelectricity of the substrate increases the wave attenuation for low values of the wave number *K*. The effect of the electromechanical coupling factor on the real part of phase velocity, *c*_1_, is remarkable for long waves and more pronounced for the layer with smaller thickness (see Figure 10a).

The influence of the piezoelectric properties of the substrate on real and imaginary parts of Love wave phase velocity for the data of materials PZT-5H—BaTiO_3_ is shown in Figure 12 and Figure 13 for electric open- and short-circuit conditions, respectively. One can observe that the electromechanical coupling factor affects the cut-off wave number. Consideration of piezoelectric properties of the substrate leads to larger values of the ‘cut-off wave number’, or in other words, the maximum wavelength at which the Love wave will propagate becomes lower under electric short circuit or open circuit conditions on the layer-vacuum interface.

## 4. Conclusions

The Love surface wave propagation in the layer with flexo- and piezo-electric properties and resting on piezoelectric substrate is investigated analytically within the strain-gradient theory. The behavior of such waves is given by the general dispersion relationship for the phase velocity dependence on the wave number. It was derived for the considered structure, taking into account the micro-inertia effect and flexoelectric properties of the guiding layer as well as the piezoelectric properties of the substrate. The physically meaningful boundary conditions are considered on the layer-substrate interface, and the layer-vacuum interface is traction free, while the electrical boundary conditions on this interface are considered as either open circuit or short circuit conditions. The derived general dispersion equation approaches to the well-known equations in literature for special cases of elastic, piezoelectric, and flexoelectric wave solutions without consideration of micro-inertia properties. The effect of flexoelectricity and the micro-inertia terms on the phase velocity is discussed in detail performing numerical parametric study with respect to material coefficients.

It is observed that effect of piezoelectric properties of the substrate on the phase wave velocity can be substantial for low values of the wave number. Numerical results indicate that both the flexoelectricity and micro-inertia have significant effect on the Love wave phase velocity. The influence of direct flexoelectricity and micro-inertia effect depends on the guiding layer thickness and becomes more significant for more narrow layers and shorter wavelengths. In general, flexoelectricity increases the Love wave phase velocity, while the micro-inertia effect decreases its value. The micro-inertia effect is more pronounced for a larger value of micro-inertia characteristic length and a smaller guiding layer thickness. The complex effect of micro-inertia terms and flexoelectricity significantly affect the profiles of dispersion curves in case of electric open circuit and/or short circuit conditions on the layer-vacuum interface. The profile of dispersion curves, as well as the presence/absence of ‘cut-off regions’ in these curves, strongly depend on the material properties of the layer and substrate, the guiding layer thickness, and the ratios between the values of flexoelectric coefficients and the micro-inertia characteristic length. For the material combination ‘PZT-5H—BaTiO_3_’ the consideration of flexoelectric properties of guiding layer leads to the appearance of the ‘cut-off wave number’ when the Love wave cannot propagate.

The flexoelectric and micro-inertia effects should not be omitted in layered structures with nanoscale dimensions. Thus, the obtained results should be useful for the design of nano-sized wave devices where high-frequency surface waves occur.

## Figures and Tables

**Table 1:** Material coefficients of the PZT-5H, BaTiO_3_, and LiNbO_3_.

Material	Mass Density ρ, $\left(10^{3}\;\mathrm{kg}/m^{3}\right)$	Elastic Constant $c_{44}$, $\left(10^{10}\;N/m^{2}\right)$	Piezoelectric Constant $e_{15}$, $\left(C/m^{2}\right)$	Dielectric Constant $a_{11}$, $\left(10^{-10}\;F/m\right)$	Shear Wave Velocity, Elastic Continuum $c_{sh}=\sqrt{c_{44}/\rho }$, $\left(10^{3}\;m/c\right)$	Shear Wave Velocity, Piezoelectric Continuum $c_{p}=\sqrt{{\bar{c}}_{44}/\rho }$, $\left(10^{3}\;m/c\right)$
*Lead Zirconate Titanate*, PZT-5H	7.5 [45]	3.53 [46]	17.0 [46]	151 [46]	2.1695	2.6942
*Barium Titanate*, BaTiO_3_	5.8 [15]	4.3 [15]	11.6 [15]	112 [15]	2.7228	3.0798
*Lithium niobate*, LiNbO_3_	4.64 [26]	6.0 [26]	3.7 [26]	3.89 [26]	3.5960	4.5294

Here, $c_{sh}=\sqrt{c_{44}/\rho }$ denotes the phase velocity of transversal waves in classical elasticity.
